# Diagnostic value of combined flow cytometry-based detection of peripheral blood lymphocyte subsets and cytokines for pediatric infectious mononucleosis

**DOI:** 10.3389/fped.2026.1836759

**Published:** 2026-06-19

**Authors:** Kai Tong, Xingran Liu, Jingxiao Dong, Qi Wang, Jing Hu, Jing Ma

**Affiliations:** Department of Laboratory Medicine, Beijing Tsinghua Changgung Hospital, School of Clinical Medicine, Tsinghua Medicine, Tsinghua University, Beijing, China

**Keywords:** cytokines, diagnostic value, Epstein–Barr virus (EBV), flow cytometry, infectious mononucleosis (IM), lymphocyte subsets

## Abstract

**Background:**

Pediatric infectious mononucleosis (IM) caused by Epstein–Barr virus (EBV) poses diagnostic challenges due to overlapping clinical presentations with other febrile illnesses. This study aimed to characterize immune function alterations in children with EBV-associated IM and evaluate the diagnostic performance of peripheral blood lymphocyte subsets and cytokine profiles, individually and in combination, to support early diagnosis.

**Methods:**

Between June 2024 and January 2026, pediatric patients with EBV-associated IM (IM group) and healthy children serving as controls (HC group) were prospectively enrolled. Lymphocyte subsets were quantified by flow cytometry, and plasma cytokines were measured by cytometric bead array (CBA). Inter-group differences were analyzed statistically, and receiver operating characteristic (ROC) curve analysis was used to evaluate diagnostic performance of individual and combined biomarkers.

**Results:**

A total of 61 children with IM and 32 healthy controls were enrolled. The predominant clinical manifestations in the IM group included fever (88.9%), pharyngitis (92.1%), lymphadenopathy (85.7%), tonsillar hypertrophy (90.4%), and periorbital edema (33.3%). Compared with the HC group, patients with IM demonstrated significantly elevated white blood cell counts, absolute lymphocyte counts, platelet counts, hemoglobin levels, and hepatic transaminases (ALT, AST) along with lactate dehydrogenase (LDH) (all *P* < 0.05). Immunophenotyping showed absolute counts of total lymphocytes, CD3⁺ T cells, CD8⁺ T cells, NK cells and percentages of CD3 and CD8 were significantly higher (*P* < 0.05) in the IM group, whereas CD4%, CD4/CD8 ratio, CD19%, and NK% were significantly decreased (*P* < 0.05). Cytokine analysis demonstrated significant elevation of IL-6, IL-10, TNF-α, IFN-*γ*, IL-17, IFN-α, and IL-12p70 in the IM group compared to the control group (*P* < 0.05). ROC analysis identified superior diagnostic performance for absolute counts of total lymphocytes (AUC=0.959), CD3⁺ T cells (AUC=0.957), and CD8⁺ T cells (AUC=0.972) among individual parameters. Combined lymphocyte subset analysis yielded an AUC of 0.995 (sensitivity 0.969, specificity 0.983), while combined cytokine assessment achieved an AUC of 0.980 (sensitivity 0.938, specificity 0.931), both substantially outperforming single biomarkers.

**Conclusions:**

Pediatric IM is characterized by profound immune activation with marked CD8⁺ T cell expansion and elevated pro-inflammatory cytokines alongside the immunoregulatory cytokine IL-10. Combined assessment of lymphocyte subsets and cytokines significantly enhances IM diagnostic accuracy and holds substantial clinical utility.

## 1 Introduction

Infectious mononucleosis (IM) is a common acute viral illness predominantly affecting children and adolescents, caused by Epstein–Barr virus (EBV) and transmitted primarily via saliva (1). The classic triad of fever, pharyngitis, and lymphadenopathy may be accompanied by tonsillar hypertrophy, hepatosplenomegaly, hepatic dysfunction, hematologic abnormalities, or immune-mediated complications (2). Due to clinical overlap with other viral and bacterial infections, early accurate diagnosis remains challenging (3).

EBV, a member of the Herpesviridae family, establishes lifelong latency within B lymphocytes following primary infection (4). Viral clearance is primarily dependent on cell-mediated immunity, particularly cytotoxic functions mediated by T lymphocytes and natural killer (NK) cells (5). During acute IM, EBV infection triggers marked immune activation, manifested as peripheral blood lymphocytosis, increased atypical lymphocytes, and profound alterations in T cell subsets (6). CD8⁺ cytotoxic T lymphocytes play a central role in controlling EBV infection, and their numerical and proportional changes correlate with disease severity and clinical outcomes (7).

Beyond lymphocyte subset changes, EBV infection induces release of numerous inflammatory and immunoregulatory cytokines, forming a complex cytokine network (8). Significant elevations of IL-6, IL-10, TNF-α, and IFN-*γ* have been documented in EBV-associated disorders, contributing to inflammatory amplification, immune modulation, and tissue injury (9, 10). Aberrant Th1/Th17-related cytokine expression during acute EBV infection is also associated with excessive immune activation and symptomatology (11). However, cytokine profiles vary across studies, and systematic characterization of cytokine dynamics and their relationship with lymphocyte subsets in pediatric IM remains limited.

Current IM diagnosis relies primarily on clinical features, complete blood count (CBC) abnormalities, EBV-specific serology, and EBV-DNA quantification (12), though these markers show limitations in early-stage or atypical cases. Flow cytometry and CBA technology now enable quantitative immune profiling, offering novel approaches to auxiliary diagnosis and disease monitoring (13). Nevertheless, individual immune parameters have restricted diagnostic utility, and optimal multi-marker integration strategies warrant further investigation (13).

Accordingly, this study systematically compared peripheral blood lymphocyte subsets and plasma cytokines between pediatric patients with IM and healthy controls, characterized immune alterations, and evaluated diagnostic performance of individual and combined biomarkers through ROC analysis, aiming to provide evidence for early IM diagnosis and improved mechanistic understanding.

## 2 Materials and methods

### 2.1 Study population

Between June 2024 and January 2026, pediatric patients diagnosed with EBV-associated IM at the outpatient and emergency departments of our institution were prospectively enrolled as the IM group. Concurrently, healthy children undergoing routine health examinations constituted the control cohort (HC group). All blood samples used in this study were residual specimens from routine complete blood count (CBC) testing. The Medical Ethics Committee of Beijing Tsinghua Changgung Hospital formally reviewed and approved the ethics application and the waiver of informed consent for this study (Ethics approval number: 26180-6-01), and authorized the use of data for publication.

#### 2.1.1 IM group inclusion and exclusion criteria

IM diagnosis was established through comprehensive clinical and laboratory evaluation (14). Inclusion criteria: (1) fulfillment of established clinical diagnostic criteria for IM; (2) positive serum EBV viral capsid antigen (VCA) IgM and/or detectable peripheral blood EBV-DNA by quantitative PCR; (3) complete clinical documentation and laboratory datasets. Exclusion criteria: (1) concurrent viral, bacterial, or fungal infection within the preceding six months; (2) underlying autoimmune disorders, immunodeficiency syndromes, hematologic diseases, or malignancies; (3) recent immunosuppressive therapy, corticosteroid administration, or interferon treatment; (4) congenital anomalies; (5) prior therapeutic interventions before enrollment.

#### 2.1.2 Eligibility criteria for healthy control group

HC group eligibility required normal physical examination findings, unremarkable laboratory parameters, and absence of recent infectious episodes.

### 2.2 Clinical data and laboratory parameter collection

Demographic data (age and sex) and IM-specific clinical manifestations (fever, pharyngitis, lymphadenopathy, tonsillar hypertrophy, periorbital edema) were systematically documented. Laboratory assessments included CBC, hepatic function panels, EBV-DNA viral load, and serum EBV-IgM antibody titers.

### 2.3 Lymphocyte subset analysis

EDTA-anticoagulated whole blood was analyzed for T/B/NK lymphocyte subset percentages and absolute counts using a Beckman Coulter DxFLEX flow cytometer with manufacturer-supplied reagents. Daily internal quality controls were implemented, and all specimens were processed within protocol-specified timeframes.

### 2.4 Plasma cytokine quantification

Four-milliliter EDTA-anticoagulated blood samples were centrifuged at 3,500 rpm for 12 min. Plasma was harvested for immediate analysis or stored at −20 °C for batch processing within one week. Twelve cytokines (IL-1β, IL-2, IL-4, IL-5, IL-6, IL-8, IL-10, IL-12p70, IL-17, TNF-α, IFN-*γ*, IFN-α) were quantified by CBA with a detection range of 0–2,500 pg/mL. Daily quality control procedures were observed.

### 2.5 Statistical analysis

Statistical analyses were performed using SPSS version 27.0. After normality assessment, parametric data were expressed as mean ± standard deviation (x̅ ± s) and compared by independent samples t-test; non-parametric variables were presented as median (interquartile range) and analyzed by Mann–Whitney U test. Categorical variables were reported as frequencies and percentages, compared by chi-square test. ROC curve analysis evaluated diagnostic performance, with area under the curve (AUC), optimal cut-off values, sensitivity, and specificity calculated. Statistical significance was defined as *P* < 0.05.

## 3 Results

### 3.1 Clinical and laboratory characteristics

A total of 61 children with IM and 32 healthy controls were enrolled. Fever occurred in 88.9% of patients with IM, pharyngitis in 92.1%, lymphadenopathy in 85.7%, tonsillar hypertrophy in 90.4%, periorbital edema in 33.3%, and hepatosplenomegaly in 16.4%. Atypical lymphocytosis (>10%) was found in 32.7% of 55 tested patients; positive plasma EBV-DNA in 78.6% of 56 tested patients; and positive serum EBV-IgM in 94.4% of 54 tested patients. Compared with the HC group, the IM group demonstrated statistically significant differences in WBC, absolute lymphocyte counts, platelet counts, hemoglobin, ALT, AST, and LDH (all *P* < 0.05), with marked leukocytosis, lymphocytosis, and hepatic dysfunction evident in a subset of patients (Table 1).

**Table 1:** Baseline clinical and laboratory characteristics of the study population.

Variable	IM group (*n* = 61)	HC group (*n* = 32)	Statistic	*P* value
Age (years)	6 (5–9.75)	9 (5.25–12)	U = 1,182.5	0.093
Sex	*χ*^2^ = 0.014			0.905
Male	37 (60.7%)	19 (59.3%)		
Female	24 (39.3%)	13 (40.6%)		
Clinical manifestations				
Pharyngitis	58 (92.1%)	—		
Lymphadenopathy	54 (85.7%)	—		
Fever	56 (88.9%)	—		
Tonsillar enlargement	57 (90.4%)	—		
Hepatosplenomegaly	10 (16.4%)	—		
Periorbital edema	21 (33.3%)	—		
Laboratory findings				
Atypical lymphocytes (>10%)	18 (32.7%)	—		
Plasma EBV-DNA positivity	44 (78.6%)	—		
EBV IgM positivity	51 (94.4%)	—		
WBC ( × 10⁹/L)	12.46 (9.63, 15.56)	6.91 (5.05, 7.08)	U = 1,727.0	**<0**.**001**
Neutrophils ( × 10⁹/L)	2.80 (1.78, 3.08)	3.01 (2.76, 4.23)	U = 876.0	0.572
Lymphocytes ( × 10⁹/L)	7.89 (5.89, 11.18)	2.07 (1.88, 3.10)	U = 1,749.0	**<0**.**001**
Platelets ( × 10⁹/L)	223.0 (184.75, 258.50)	292.0 (253.00, 335.00)	U = 476.0	**<0**.**001**
Hemoglobin (g/L)	127.0 (116.00, 132.25)	131.0 (121.00, 140.00)	U = 641.5	**0**.**016**
ALT (U/L)	45.55 (25.17, 137.15)	15.90 (12.00, 17.20)	U = 1,235.0	**<0**.**001**
AST (U/L)	45.45 (34.75, 83.57)	25.70 (16.80, 32.70)	U = 1,222.5	**<0**.**001**
LDH (U/L)	425.85 (325.67, 499.75)	220.00 (194.00, 249.00)	U = 994.5	**<0**.**001**

Data are expressed as median (IQR) or *n* (%). *P* < 0.05 was considered statistically significant. ALT, alanine aminotransferase; AST, aspartate aminotransferase; EBV, Epstein–Barr virus; HC, healthy control; IM, infectious mononucleosis; IQR, interquartile range; LDH, lactate dehydrogenase; WBC, white blood cell count.

Bold values indicate statistically significant differences between groups (*P* < 0.05).

### 3.2 Lymphocyte subset profiles

Relative to the HC group, the IM group exhibited significantly elevated absolute counts of total lymphocytes, NK cells, CD3⁺ T cells, CD4⁺ T cells, and CD8⁺ T cells (all *P* < 0.05), with correspondingly increased CD3% and CD8%. Conversely, CD19%, NK%, CD4%, and the CD4/CD8 ratio were markedly reduced (all *P* < 0.05) (Table 2). Notably, although CD19% was significantly decreased in the IM group, the absolute CD19⁺ B cell count was not significantly different between groups (*P* = 0.768), reflecting a relative dilution effect due to the massive expansion of CD8⁺ T cells.

**Table 2:** Comparison of lymphocyte subset parameters between the IM group and HC group.

Parameter	IM group (*N* = 61)	HC group (*n* = 32)	U value	*P* value
Lym (cells/*μ*L)	8,031.16 (5,527.63, 11,487.92)	2,249.40 (1,754.00, 2,926.07)	109.00	**<0**.**001**
CD19 (%)	3.23 (2.52, 4.71)	15.05 (10.63, 17.51)	1,756.00	**<0**.**001**
CD19 (cells/μL)	328.45 (187.60, 462.65)	317.15 (228.54, 387.12)	963.00	0.768
NK (%)	8.01 (6.25, 10.62)	12.47 (6.80, 15.52)	1,252.00	**0**.**017**
NK (cells/μL)	733.35 (420.78, 1,031.77)	237.53 (173.87, 341.43)	258.00	**<0**.**001**
CD3 (%)	85.60 (83.67, 88.84)	71.85 (64.67, 76.53)	181.00	**<0**.**001**
CD3 (cells/μL)	7,032.31 (4,407.32, 10,231.93)	1,687.30 (1,206.78, 2,052.45)	79.00	**<0**.**001**
CD4 (%)	12.95 (9.28, 19.73)	35.02 (30.41, 38.82)	1,807.00	**<0**.**001**
CD4 (cells/μL)	1,039.66 (776.99, 1,497.68)	787.75 (549.98, 1,149.95)	569.00	**0**.**002**
CD8 (%)	64.58 (50.97, 68.81)	26.83 (22.46, 31.51)	129.00	**<0**.**001**
CD8 (cells/μL)	5,086.32 (3,487.11, 7,832.74)	640.38 (462.31, 840.84)	52.00	**<0**.**001**
CD4/CD8 ratio	0.18 (0.14, 0.43)	1.33 (1.11, 1.58)	1,827.50	**<0**.**001**
CD4⁻CD8⁻ (%)	5.87 (0.89, 11.06)	6.84 (5.56, 10.23)	1,127.00	**0**.**066**

Data are expressed as median (IQR). *P* < 0.05 was considered statistically significant.

HC, healthy control; IM, infectious mononucleosis; IQR, interquartile range; Lym, total lymphocyte absolute count; NK, natural killer cell.

Bold values indicate statistically significant differences between groups (*P* < 0.05).

### 3.3 Cytokine expression profiles

Plasma concentrations of IL-6, IL-10, TNF-α, IFN-*γ*, IL-17, IFN-α, and IL-12p70 were significantly elevated in the IM group compared with HC subjects (all *P* < 0.05), indicating pronounced immune activation. IL-2, IL-4, IL-5, IL-8, and IL-1β showed no significant inter-group differences (Table 3).

**Table 3:** Comparison of plasma cytokine levels between the IM group and HC group.

Parameter (pg/mL)	IM group (*N* = 61)	HC group (*n* = 32)	U value	*P* value
IL-2	1.12 (0.64, 15.61)	0.89 (0.80, 0.99)	713.00	0.070
IL-4	1.39 (0.96, 2.11)	1.50 (1.35, 1.59)	1,036.00	0.363
IL-6	5.94 (3.32, 11.21)	2.59 (2.23, 3.17)	437.00	**<0**.**001**
IL-10	8.26 (2.27, 21.43)	3.96 (2.85, 5.19)	694.00	**0**.**049**
TNF-α	4.01 (2.29, 16.26)	1.63 (1.22, 2.30)	372.00	**<0**.**001**
IFN-*γ*	2.63 (1.97, 5.06)	1.48 (1.15, 2.43)	449.00	**<0**.**001**
IL-17	2.30 (1.33, 3.24)	1.43 (1.20, 1.68)	466.00	**<0**.**001**
IL-8	9.35 (1.14, 31.82)	13.02 (7.80, 21.59)	1,060.00	0.266
IFN-α	2.31 (1.59, 3.44)	1.09 (0.73, 1.58)	229.00	**<0**.**001**
IL-12p70	2.00 (1.09, 2.98)	1.01 (0.72, 1.40)	425.00	**<0**.**001**
IL-5	1.25 (0.91, 1.85)	1.16 (1.02, 1.40)	863.00	0.584
IL-1β	2.31 (1.53, 4.46)	3.12 (2.56, 5.21)	1,161.00	0.050

Data are expressed as median (IQR). *P* < 0.05 was considered statistically significant.

HC, healthy control; IFN, interferon; IL, interleukin; IM, infectious mononucleosis; IQR, interquartile range; TNF-α, tumor necrosis factor-alpha.

Bold values indicate statistically significant differences between groups (*P* < 0.05).

### 3.4 Diagnostic performance of immune biomarkers

#### 3.4.1 Lymphocyte subset ROC analysis

**Figure 1:**
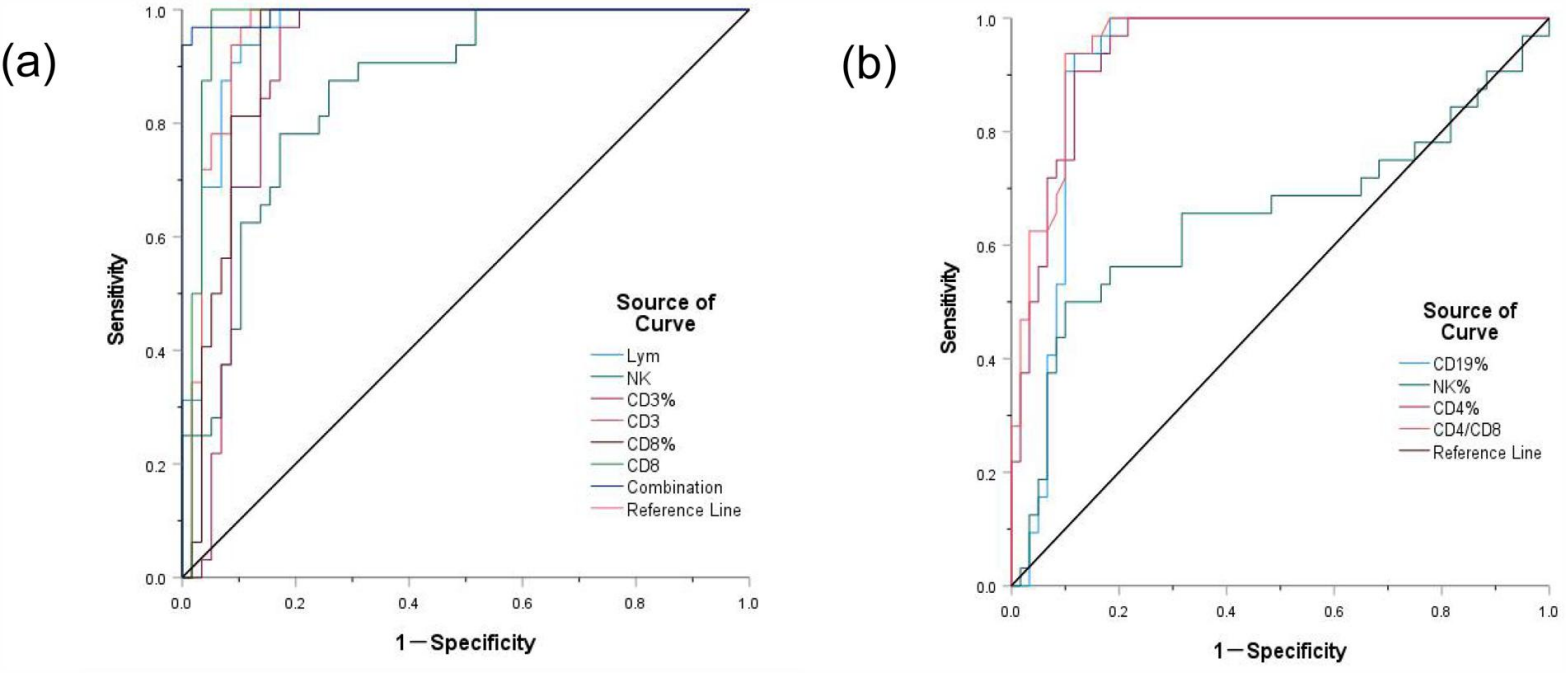
ROC curve of metrics and combined detection metrics in differentiating IM caused by EBV infection. **(a)** The ROC curves of absolute lymphocyte count (LYM), NK cell count (NK), CD3+ T cell percentage (CD3%), CD3+ T cell count (CD3), CD8+ T cell percentage (CD8%), CD8+ T cell count (CD8), and their combination in differentiating IM Caused by EBV Infection. **(b)** The ROC curves of the percentages of lymphocyte subsets (CD3+ T cell percentage [CD3%], CD4+ T cell percentage [CD4%], CD8+ T cell percentage [CD8%], CD19+ B cell percentage [CD19%], NK cell percentage [NK%]), absolute counts (lymphocyte count [LYM], NK cell count [NK], CD3+ T cell count [CD3], CD8+ T cell count [CD8]), the CD4/CD8 ratio, and their combination in differentiating IM Caused by EBV Infection.

Among lymphocyte subset parameters, CD8⁺ T cell count yielded the highest individual AUC (0.972; 95% CI 0.934–1.000), followed by total lymphocyte count (AUC = 0.959) and CD3⁺ T cell count (AUC = 0.957). Combined lymphocyte subset analysis achieved an AUC of 0.995 (95% CI 0.984–1.000; sensitivity 96.9%; specificity 98.3%), substantially outperforming all individual markers (Table 4, Figure 1).

**Table 4:** Diagnostic performance of lymphocyte subset parameters for distinguishing IM from healthy controls.

Parameter	AUC	95% CI	*P* value	Cut-off	SE	SP	YI	PPV	NPV
Lym (cells/μL)	0.959	(0.920, 0.997)	**0**.000**	3,867.33	0.938	0.897	0.835	0.937	0.881
CD3 (%)	0.902	(0.835, 0.970)	**0**.000**	81.33	0.969	0.828	0.797	0.915	0.933
CD3 (cells/μL)	0.957	(0.914, 1.000)	**0**.000**	3,015.35	1.000	0.879	0.879	0.940	1.000
CD4 (%)	0.941	(0.896, 0.986)	**0**.000**	28.27	0.906	0.883	0.789	0.937	0.831
CD8 (%)	0.930	(0.875, 0.986)	**0**.000**	39.69	1.000	0.862	0.862	0.931	1.000
CD8 (cells/μL)	0.972	(0.934, 1.000)	**0**.000**	1,085.99	1.000	0.948	0.948	0.973	1.000
CD4/CD8 ratio	0.952	(0.912, 0.992)	**0**.000**	0.83	0.938	0.900	0.838	0.947	0.884
CD19 (%)	0.915	(0.851, 0.979)	**0**.000**	8.75	0.938	0.883	0.821	0.939	0.882
NK (%)	0.652	(0.520, 0.784)	**0**.000**	12.36	0.500	0.900	0.400	0.905	0.486
NK (cells/μL)	0.861	(0.786, 0.936)	**0**.000**	473.13	0.875	0.741	0.616	0.866	0.757
Combined	0.995	(0.984, 1.000)	**0**.000**	—	0.969	0.983	0.952	0.991	0.943

AUC, area under the curve; 95% CI, 95% confidence interval; Cut-off, optimal cutoff value; NPV, negative predictive value; PPV, positive predictive value; SE, sensitivity; SP, specificity; YI, Youden index. *P* value indicates comparison of the AUC of the ROC curve against the null value of 0.5.

** *P* < 0.01.

#### 3.4.2 Cytokine ROC analysis

**Figure 2:**
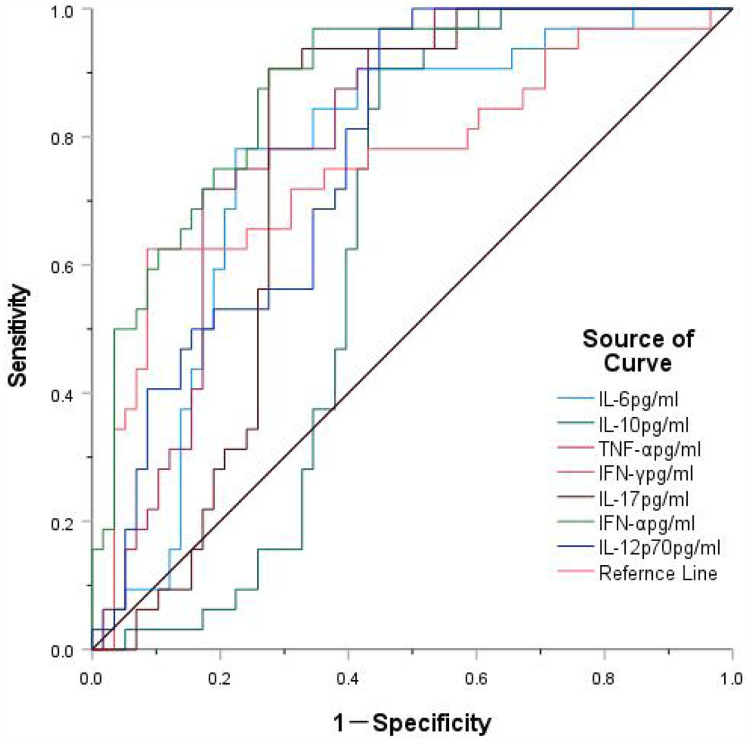
The ROC curves of interleukin-6 (IL-6), interleukin-10 (IL-10), tumor necrosis factor-alpha (TNF-α), interferon-gamma (IFN-*γ*), interleukin-17 (IL-17), interleukin-12p70 (IL-12p70), interferon-alpha (IFN-α), and their combination in differentiating IM caused by EBV infection.

Among individual cytokines, IFN-α exhibited the highest diagnostic efficacy (AUC = 0.877; 95% CI 0.806–0.947), followed by TNF-α (AUC = 0.800) and IL-12p70 (AUC = 0.771). The combined cytokine panel achieved an AUC of 0.980 (95% CI 0.957–1.000; sensitivity 93.8%; specificity 93.1%), markedly exceeding single-cytokine performance (Table 5, Figure 2).

**Table 5:** Diagnostic performance of cytokine parameters for distinguishing IM from healthy controls.

Parameter	AUC	95% CI	*P* value	Cut-off (pg/mL)	SE	SP	YI	PPV	NPV
IL-6	0.765	(0.663, 0.866)	**0**.000**	3.208	0.781	0.776	0.554	0.869	0.650
IL-10	0.625	(0.511, 0.742)	**0**.049*	7.153	0.906	0.552	0.458	0.794	0.755
TNF-α	0.800	(0.709, 0.890)	**0**.000**	1.886	0.719	0.828	0.547	0.888	0.607
IFN-γ	0.758	(0.648, 0.869)	**0**.000**	1.755	0.625	0.914	0.539	0.923	0.561
IL-17	0.749	(0.646, 0.852)	**0**.000**	1.765	0.906	0.724	0.630	0.862	0.802
IFN-α	0.877	(0.806, 0.947)	**0**.000**	1.705	0.906	0.724	0.630	0.862	0.802
IL-12p70	0.771	(0.676, 0.866)	**0**.000**	1.795	0.969	0.552	0.521	0.804	0.903
Combined	0.980	(0.957, 1.000)	**0**.000**	—	0.938	0.931	0.869	0.963	0.887

AUC, area under the curve; 95% CI, 95% confidence interval; Cut-off, optimal cutoff value (pg/mL); NPV, negative predictive value; PPV, positive predictive value; SE, sensitivity; SP, specificity; YI, Youden index. *P* value indicates comparison of the AUC of the ROC curve against the null value of 0.5.

Bold values indicate statistically significant differences between groups (*P* < 0.05).

* *P* < 0.05.

** *P* < 0.01.

## 4 Discussion

This study provides comprehensive characterization of peripheral blood immune alterations in pediatric EBV-associated IM, demonstrating that profound changes in lymphocyte subset distribution and cytokine profiles are closely linked to immune activation status, and that combined immune biomarker analysis significantly enhances diagnostic precision.

Regarding lymphocyte dynamics, we observed marked expansion of CD8⁺ T cell absolute counts and proportions, alongside relative reductions in CD4⁺ T cells, CD19⁺ B cells, and NK cell percentages, indicating robust cellular immune activation following EBV infection. These findings align with prior reports demonstrating peripheral expansion of CD3⁺ and CD8⁺ T populations with concurrent diminution of CD4⁺ T and B cell frequencies in pediatric IM (15). This pattern reflects the fundamental biology of EBV infection: the virus primarily targets B lymphocytes, subsequently triggering EBV-specific CD8⁺ T cell-mediated clearance that drives pronounced effector expansion during acute infection (16). EBV-infected B cells present viral peptides via MHC class I molecules, activating naïve CD8⁺ T cells and promoting their differentiation into cytotoxic T lymphocytes (CTL) (6). These CTLs eliminate infected cells through perforin and granzyme B, while concurrently secreting IFN-*γ* to amplify antiviral immunity (5). Notably, excessive CD8⁺ T cell activation may also contribute to immunopathological tissue injury, particularly in severe EBV-associated complications such as hemophagocytic lymphohistiocytosis (HLH) (22). Lymphocyte proportions typically normalize with disease resolution, underscoring the dynamic and reversible nature of immune activation (15). Upregulation of inhibitory checkpoint molecules including PD-1 and CTLA-4 on T cell surfaces suggests a negative feedback mechanism limiting excessive inflammation (15, 21), and elevated granzyme B production by CD8⁺ T and NK cells further highlights the central role of cellular immunity in antiviral defense (17).

Regarding B cell dynamics, the maintained absolute CD19⁺ B-cell count alongside a substantial decline in CD19⁺ percentage indicates a relative dilution effect. Robust expansion of CD8⁺ T cells elevates the total lymphocyte count, thereby lowering the relative proportion of B cells without inducing genuine absolute B-cell depletion. Concurrent redistribution of EBV-activated B cells toward secondary lymphoid organs may further account for this discrepancy. These findings highlight that percentage-based and absolute count-based lymphocyte parameters offer complementary rather than overlapping diagnostic implications. Clinically, relative and absolute lymphocyte indices should therefore be interpreted in combination.

The prominent CD8⁺ T cell expansion and cytokine activation in our cohort bears resemblance to EBV-associated HLH immunology, though critical distinctions exist. HLH is characterized by dysregulated, uncontrolled immune activation culminating in cytokine storm and multi-organ failure (23, 26). While both conditions feature CD8⁺ T cell expansion and elevated pro-inflammatory cytokines (24), HLH demonstrates substantially higher cytokine concentrations with extreme soluble IL-2 receptor (sCD25) and ferritin elevations, alongside profound NK cell dysfunction (25). These observations suggest that IM and HLH represent distinct stages along the EBV immune response spectrum: IM manifests as controlled, self-limited activation, whereas HLH reflects pathological hyperactivation after regulatory mechanism failure (27). Serial monitoring of CD8⁺ T cell levels and cytokine profiles may therefore facilitate early identification of patients at risk of progressing from IM to HLH.

Our cytokine profiling revealed significant elevation of IL-6, IL-10, TNF-α, IFN-*γ*, IL-17, IFN-α, and IL-12p70 in patients with IM, alongside preserved levels of IL-2, IL-4, IL-5, IL-8, and IL-1β. Multiple studies have documented increased pro-inflammatory and immunoregulatory cytokines during EBV infection (10, 18). IFN-*γ* promotes CD8⁺ T cell antiviral function, upregulates MHC class I expression, and synergizes with TNF-α to activate macrophages (28). IL-6 orchestrates acute-phase responses and participates in lymphocyte activation and differentiation (29). IL-12p70 is a critical innate-to-adaptive immunity bridge cytokine produced by dendritic cells and macrophages upon recognition of viral pathogens; during EBV infection, IL-12 drives differentiation of naïve T cells toward a Th1 phenotype and potentiates cytotoxic function of NK cells and CD8⁺ T lymphocytes (32), directly contributing to the marked CD8⁺ T cell expansion observed in our cohort. Elevated IL-17, a hallmark Th17 effector cytokine, reflects activation of the Th17 axis during acute EBV infection (33). Th17-mediated responses contribute to local mucosal inflammation and may underlie symptoms such as pharyngitis and lymphadenopathy. The concurrent elevation of both Th1 cytokines (IFN-*γ*, IL-12p70, TNF-α) and the Th17 cytokine IL-17 suggests a broad, multi-lineage T helper response rather than a strictly polarized Th1 reaction, consistent with the complex immunopathology of acute EBV infection.

Regarding IL-10, it is important to clarify that this cytokine is a key immunoregulatory rather than pro-inflammatory mediator. Its significant elevation in children with IM likely reflects two concurrent mechanisms. First, IL-10 is produced by regulatory T cells, macrophages, exhausted CD8⁺ T cells, and activated B cells as a compensatory response to contain the intense immune activation during acute EBV infection, thereby limiting immunopathological tissue damage (35). Second, EBV encodes a structural homolog of human IL-10 (viral IL-10, vIL-10), encoded by the BCRF1 gene, which retains most immunosuppressive functions of human IL-10 (34). This vIL-10 suppresses antiviral Th1 responses, inhibits cytokine production by monocytes and macrophages, and facilitates immune evasion by EBV. Plasma IL-10 elevation in patients with IM therefore represents both a host-protective mechanism limiting immunopathology and an EBV-driven immune evasion strategy. These cytokine elevations indicate pronounced inflammatory activation in patients with IM, likely underlying high-grade fever and lymphadenopathy (19). The balance between pro-inflammatory and immunoregulatory responses critically influences symptom emergence and resolution.

Post-EBV immune responses exhibit distinct temporal phases. During acute infection (1–2 weeks), CD8⁺ T cells expand rapidly, cytokines surge, and characteristic symptoms manifest as the immune system mobilizes intensively (5). During convalescence (2–4 weeks), CD8⁺ T cell numbers contract, cytokine levels decline, and symptoms resolve through effective immunoregulatory mechanisms (30). However, in a minority of patients, regulatory failure or excessive viral burden may enable progression from self-limited IM to chronic active EBV infection (CAEBV) or HLH (31). Initial CD8⁺ T cell activation intensity, cytotoxic function, and regulatory T cell (Treg) suppressive capacity appear to influence disease trajectory (22). Thus, the immune activation profile observed in our cohort likely represents an early, controllable disease phase, and continuous monitoring could enable outcome prediction and high-risk population identification.

Our ROC analysis demonstrated variable diagnostic performance among individual biomarkers, while multi-marker integration substantially enhanced accuracy. Among lymphocyte subset parameters, CD8⁺ T cell absolute count achieved the highest individual AUC (0.972), and the combined lymphocyte subset panel reached an AUC of 0.995 with sensitivity and specificity both exceeding 96%, suggesting that integrated immune profiling more comprehensively captures systemic immune status during EBV infection. Regarding cytokine diagnostic performance, IFN-α showed the highest individual AUC (0.877), reflecting its role as a frontline innate antiviral mediator induced rapidly during EBV infection, while the combined cytokine panel achieved an AUC of 0.980 (sensitivity 93.8%, specificity 93.1%). These findings are broadly consistent with previous reports: Zhang et al. (20) documented significant elevation of inflammatory cytokines in pediatric patients with IM, and Wang et al. (9) similarly characterized immune dysregulation in EBV-associated IM. However, direct quantitative ROC comparisons across studies remain limited by heterogeneity in cytokine panels, analytical platforms, and reference populations. Collectively, the superior performance of both combined panels demonstrates that multi-marker integration more comprehensively captures the systemic immune remodeling characteristic of EBV infection and is superior to single-parameter approaches in clinical practice.

This study has several limitations. First, the single-center prospective design with relatively modest sample size necessitates validation through larger, multicenter studies. Second, the control group comprised only healthy children, without disease controls (e.g., other febrile illnesses), which may limit specificity assessment. Third, local lymphoid tissue immune responses may influence peripheral immune parameters, warranting further investigation. Finally, different EBV subtypes or concurrent viral reactivation may modulate immune response patterns, meriting future analysis.

## 5 Conclusion

Using flow cytometry and CBA technology, this study systematically characterized peripheral blood lymphocyte subsets and cytokine profiles in pediatric patients with IM. Results demonstrated significant immune cell remodeling and inflammatory mediator dysregulation, principally manifesting as expanded CD8⁺ T cell populations and elevated pro-inflammatory cytokines alongside the immunoregulatory cytokine IL-10. Combined assessment of lymphocyte subsets and cytokine profiles significantly enhances IM diagnostic accuracy and provides valuable mechanistic insights. These findings offer evidence-based support for early IM identification and lay groundwork for future immunological investigations in larger, multicenter cohorts.

## Data Availability

The original contributions presented in the study are included in the article/Supplementary Material, further inquiries can be directed to the corresponding author.
